# Functional Redundancy in Soil Microbial Community Based on Metagenomics Across the Globe

**DOI:** 10.3389/fmicb.2022.878978

**Published:** 2022-05-02

**Authors:** Huaihai Chen, Kayan Ma, Caiyan Lu, Qi Fu, Yingbo Qiu, Jiayi Zhao, Yu Huang, Yuchun Yang, Christopher W. Schadt, Hao Chen

**Affiliations:** ^1^State Key Laboratory of Biocontrol, School of Ecology, Sun Yat-sen University, Shenzhen, China; ^2^Key Laboratory of Pollution Ecology and Environment Engineering, Institute of Applied Ecology, Chinese Academy of Sciences, Shenyang, China; ^3^Key Lab of Conservation Tillage and Ecological Agriculture, Shenyang, China; ^4^Oak Ridge National Laboratory, Biosciences Division, Oak Ridge, TN, United States

**Keywords:** functional redundancy, soil metagenomics, functional traits, taxonomic compositions, latitude

## Abstract

Understanding the contribution of soil microbial communities to ecosystem processes is critical for predicting terrestrial ecosystem feedbacks under changing climate. Our current understanding lacks a consistent strategy to formulate the linkage between microbial systems and ecosystem processes due to the presumption of functional redundancy in soil microbes. Here we present a global soil microbial metagenomic analysis to generalize patterns of microbial taxonomic compositions and functional potentials across climate and geochemical gradient. Our analyses show that soil microbial taxonomic composition varies widely in response to climate and soil physicochemical gradients, while microbial functional attributes based on metagenomic gene abundances are redundant. Among 17 climate zones, microbial taxonomic compositions were more distinct than functional potentials, as climate and edaphic properties showed more significant influence on microbial taxonomic compositions than on functional potentials. Microbial taxonomies formed a larger and more complex co-occurrence network with more module structures than functional potentials. Functional network was strongly inter-connected among different categories, whereas taxonomic network was more positively interactive in the same taxonomic groups. This study provides strong evidence to support the hypothesis of functional redundancy in soil microbes, as microbial taxonomic compositions vary to a larger extent than functional potentials based on metagenomic gene abundances in terrestrial ecosystems across the globe.

## Introduction

Soil microbial communities play a major role in mediating biogeochemical cycling of carbon (C) and nutrients, and thus in maintaining ecosystem functions. With the increasing concern of climate change, microbes’ ability to respond to climate change will likely be key to the maintenance of critical functions in ecosystems across the globe (Cavicchioli et al., 2019). Although linking microorganisms with biogeochemical cycles is crucial for understanding such maintenance process, we still don’t know the suitable resolution for formulating the connection between microbial diversity and ecosystem processes such that microbes’ taxonomic distributions, metabolic capabilities and ability to respond to environmental perturbations can be captured. While microbes are incredibly diverse (Gans et al., 2005), the majority of biogeochemical transformations appear to be mediated by a limited set of metabolic pathways across a variety of taxonomic groups (Louca et al., 2018). Accordingly, studies on microbial populations have often found a high sensitivity of microbial taxonomic composition to external perturbations (Allison and Martiny, 2008), while microbial functions often remain relatively stable (Langenheder et al., 2005; Louca et al., 2016). Such decoupling of microbial functions from taxonomic composition raises critical questions regarding the relative value of microbial phylogenetic diversity vs. functional microbial metabolic diversity information across space and time for understanding biogeochemical responses.

Microbial taxonomic composition and functional potential may not be always linearly associated. Globally, soil microbial communities are typically dominated by a small subset of phylotypes, though their biogeochemical functions are not dissimilar to the other phylotypes (Delgado-Baquerizo et al., 2018). Additionally, though bacterial phylotypes sharing similar edaphic habitats tend to co-occur (Bahram et al., 2018; Delgado-Baquerizo et al., 2018), the identity of the microbes does not necessarily determine the microbial metabolic functions in each community. As a broad range of taxa can potentially perform similar metabolic functions, and therefore compositional shifts of microbial community do not always alter ecosystem processes, functional redundancy has thus been implicated as a prevailing phenomenon across microbial communities (Louca et al., 2018). For instance, algal-associated bacterial communities showed a high phylogenetic variability in species composition, but their metagenomic functional potentials are relatively stable (Burke et al., 2011). Using the16S rRNA amplicon sequencing and GeoChip technologies, it has been shown that the soil bacterial potential function is highly convergent along the latitudinal gradient with highly differing bacterial community compositions (Zhang et al., 2016). In the soil, not only bacteria but also fungi have been found endemic to particular bioregions with functional convergence of extracellular enzyme activity across the North American (Talbot et al., 2014). Soil manipulation experiments have shown that shifts of microbial community composition *via* serial dilution have little effect on the functioning of microcosms (Wertz et al., 2007). However, similar dilution-to-extinction experiments have also revealed the depletion of specific enzyme activities in response to reduced microbial diversity (Peter et al., 2011). Thus, careful assessment of the composition of multiple functions is required to better understand the relationship between microbial diversity and ecosystem function in soils (Bastida et al., 2016; Delgado-Baquerizo et al., 2016).

Prediction of functional characteristics in ecosystems usually rely on using environmental covariates as proxy of microbial activity to drive biogeochemical transformations, while representations of the composition and specific metabolic functions of microbial communities are often absent. However, unifying global patterns in soil microbes and generalizing microbial diversity and functional potentials have traditionally been obscured by the local variability of soil communities. Current studies are increasingly deploying metagenomics-based approaches as a promising tool (Tringe et al., 2005) to study the relationships between functional and taxonomic diversities (Fierer et al., 2012, 2013; Pan et al., 2014; Leff et al., 2015; Souza et al., 2015). For example, a recent meta-analysis based on a metagenomic dataset of 365 samples obtained across the globe revealed consistent patterns of the relative frequency of eight metabolic pathways associated with nitrogen (N) transformations, hinting at the ability of metagenomic approaches to make potential inferences regarding the microbial role in mediating biogeochemical cycling (Nelson et al., 2016). Similarly, a recent analysis of soil microbial metagenomes from 18 biomes across the globe demonstrated the redox gene sets in microbial communities are distinct among biomes (Ramírez-Flandes et al., 2019). The growing wealth of soil metagenome data thus seems poised well to aid in the generalization of global patterns of microbial attributes and standardization of frameworks for consistent representation of microbial community.

Here, we collected and synthesized soil metagenomes across the globe that are publicly available from peer-reviewed publications to investigate the general patterns of microbial communities and their functional potentials. It should be noted that we did not directly measure soil microbial functions. Instead, we used microbial functional composition based on metagenomically-derived gene abundances to represent microbial functional potentials. In this study, we assumed that the abundance of each functional gene can be specific to a particular microbial enzyme-catalyzed processes, so numerous microbially-mediated functional potentials can be examined all together in one soil metagenomic sample. Here, we hypothesize that there is a general microbial functional redundancy instead of taxonomic redundancy in global terrestrial ecosystems, which can be evidenced if the diversity of microbial taxonomy is significantly higher than functional potentials derived from metagenomic genes. We understood that inferring microbial functioning from metagenomic genes may have weakness. For example, the relative abundance of functional genes may remain stable despite the microbial functional activities significantly vary. In addition, the shifts of relative abundance of typical genes may be simply due to the interaction between these genes and the rest of the metagenome. However, the objective of this metagenomic analysis is to understand the generalized patterns and linkages of microbial taxonomic compositions and functional potentials derived from metagenomic data. By unifying the patterns of these relationships within the microbial community, we can provide metagenomic evidence to test the hypothesis of functional redundancy in soil microbes based on metagenomic gene abundances in terrestrial ecosystems across the globe.

## Materials and Methods

### Data Collection

**FIGURE 1:**
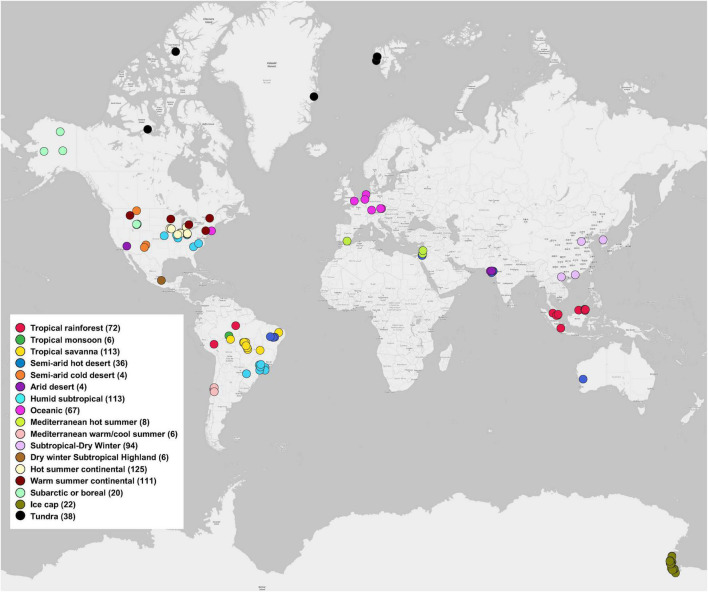
Global distribution of 845 soil metagenomes. Locations are grouped by 17 climate zones from 51 publications used in this study. Sample sizes of each climate zone classification are given in parentheses.

We used the MG-RAST server, because the metagenomes data deposited in the server are open and accessible publicly. In addition, the server contains functional analysis and can directly export functional and taxonomic composition matrices from metagenomic data. Instead of directly obtaining available shotgun metagenomic data from MG-RAST server, we only selected soil metagenomes that have been published in peer-reviewed journals to ensure the quality and completeness of the metagenomic data, because we believed that the peer-review process in publication papers can confer a tighter control over the quality of the metagenomic data that have been included in our study. We searched peer-reviewed publications from 2012 to 2018 using the Web of Science database for our literature survey on December 10, 2018. Search keyword combinations included “soil, metagenome, MG-RAST,” “soil, shotgun sequencing, MG-RAST,” and “soil, Illumina, MG-RAST.” The following criteria were applied: (1) studies that analyzed and/or directly deposited soil metagenomes (generated using shotgun sequencing that involves randomly breaking the DNA into a collection of small fragments that are sequenced individually to obtain reads) to the MG-RAST database; (2) soil metagenomes in the MG-RAST database that could be publicly accessible. Based on the Study ID and/or the MG-RAST ID reported in the publications, we further extracted data matrix of soil metagenomes from the MG-RAST database (Chen et al., 2021a,b). In total, this study included 845 soil metagenomes across 17 climate zones around the world, extracted from 56 MG-RAST studies published in 51 peer-reviewed papers (Figure 1). Details of each soil metagenome extracted from publications and the MG-RAST database was given in Supplementary Table 1. For example, Study ID, MG-RAST ID, sample name, bp (base pair), and reads were obtained from metadata of each soil metagenome in MG-RAST database. Generally, the ranges (mean ± standard error) of bp (base pair), raw reads, SEED Subsystems hits, and RefSeq hits were 2.41×10^9^ ± 1.34×10^8^, 2.05×10^7^ ± 1.34E×10^6^, 2.01×10^6^ ± 1.22×10^5^, and 5.11×10^6^ ± 2.96×10^5^, respectively.

For each soil metagenome, using the “Analysis” function of the MG-RAST server, we loaded the SEED Subsystems database, which contain functional information structured into three levels, with Level 1 being the most general class while the Level 3 being the most specific class (Function) (Overbeek et al., 2013), as the metagenomic functional potentials, and loaded from the RefSeq database taxonomic information at genus, family, order, class, and phylum levels (Taxonomy) (Tatusova et al., 2013) as taxonomic compositions. Although RefSeq database had taxonomic resolution to species, it was not recommended by the server for most application using shotgun sequence data to infer taxonomic information below the genus level, so we kept the lowest taxonomic information at the genus level in this study.

The analyses were performed using default settings (maximum *e*-value cutoff was 1e^–5^, minimum identity cutoff was 60%, and minimum alignment length was 50) (Meyer et al., 2008). We further merged data matrix of each functional or taxonomic group extracted from different studies together to generate new datasets of microbial profiles of functional potentials annotated by the SEED Subsystems database and taxonomic compositions annotated by the RefSeq database. We chose Subsystems database for functional grouping rather than the KEGG Orthology (KO) (Kanehisa et al., 2015), Clusters of Orthologous Groups (COG) (Galperin et al., 2014), and Non-supervised Orthologous Groups (NOG) (Huerta-Cepas et al., 2015) databases because Subsystems had more than 21 classification at Level 1, comparable to RefSeq taxonomy at phylum levels, allowing us to conduct comparison between taxonomic and functional annotations. We chose RefSeq database rather than the traditional ribosomal RNA databases, such as RDP (Ribosomal Database Project) (Cole et al., 2008), Greengenes (Desantis et al., 2006), or Silva LSU/SSU (Pruesse et al., 2007) databases, because taxonomic hits in the RefSeq database were over 1,000-fold higher than the rRNA databases, rendering the resolution comparable to functional hits for comparison (Supplementary Table 1). Since the assembled and unassembled sequences may result in potential bias in the diversity of taxonomy and function annotations, all the publication included in this study have deposited or submitted their unassembled raw sequences in MG-RAST server. Even though some studies reported their own processing data based on *de novo* assembled contigs in their publications, as long as they used MG-RAST server to deposit their unassembled raw sequences, they were all included in this study to increase the coverage of our datasets.

The metagenomic data deposited in the MG-RAST server were either assembled or unassembled sequences, both of which were included in this study to increase the coverage of our datasets.

The geographic coordinates of latitudes (LAT) and longitudes (LONG) of each soil metagenome were directly obtained from publications. Based on LAT and LONG, climate data of mean annual temperature (MAT) and precipitation (MAP) of the study sites for each soil metagenome were extracted from the WorldClim version 2 dataset (Fick and Hijmans, 2017) using the R package “raster.” Because not all study provided edaphic property data, we obtained soil properties, including C (soil total carbon content, g C kg-1 soil), pH (soil pH measured in a 1:5 w/v suspension of deionized water, no unit), sand (percentage of soil particles ranging from 2.0 to 0.05 mm in diameter, %), silt (percentage of soil particles ranging from 0.05 to 0.002 mm in diameter, %), and clay (percentage of soil particles smaller than 0.002 mm in diameter, %), from the SoilGrids database^1^ (Hengl et al., 2017). Typically, the model data for climate and edaphic properties were used to compare their correlation with functional and taxonomic diversities of each soil metagenome tested in our study. To examine how microbial taxonomic and functional diversities differ globally, soil metagenomic data was classified into 17 climate zones based on the main classification of Koeppen-Geiger Climatic Zones (Kottek et al., 2006) using the R package “kgc.”

### Statistical Analyses

To minimize bias caused by different sequencing depths and read lengths among studies, we standardize the hit of each functional or taxonomic category in the data matrix to relative abundance through dividing by total hits to calculate beta-diversity of all tested samples. We found the functional and taxonomic diversities among different samples did not depend on the sequencing depths and read lengths of the tested samples. Even if the size of the dataset might affect the direct comparison among soil metagenomes, this study mainly focused on assessment of the relationships between functional and taxonomic diversities as well as climate and soil distances. Thus, the size of the dataset should have similar overall effects on both functional and taxonomic compositions in the same soil metagenomes, which would not lead to significant bias as a result. Based on the relative functional abundance at level 3 (Function, 1,135 categories) and the taxonomic abundance at genus level (Taxonomy, 1,113 categories), we calculated Bray-Curtis similarity and constructed triangular pairwise Bray-Curtis similarity matrices, which were further transformed to lists of pairwise Bray-Curtis similarities ordered by sample names in PRIMER 7 (Plymouth Routines in Multivariate Ecological Research Statistical Software, v7.0.13, PRIMER-E Ltd., United States) (Clarke and Gorley, 2015). Pearson’s correlation between these Bray-Curtis matrices of functional and taxonomic similarity were constructed to examine the relationship between functional and taxonomic diversities. Based on the MAT and MAP data (Climate) and soil properties of C, pH, sand, silt, and clay (Soil), triangular pairwise Euclidean distance was calculated to construct triangular pairwise Euclidean distance matrix, which were further transformed to a list of pairwise Euclidean distance ordered by sample names in PRIMER 7. To evaluate how functional and taxonomic similarity decays with climate and soil distance, Pearson’s correlations were constructed between the pairwise Euclidean distance of climate and soil data and Bray-Curtis similarity of function and taxonomy. To analyze functional and taxonomic composition structures of soil metagenomes annotated in Subsystems database at Level 3 (Function) and in RefSeq database at genus level (Taxonomy), PCoA (principal coordinates analysis), and PERMANOVA (Permutational multivariate analysis of variance) were conducted using the triangular pairwise Bray-Curtis similarity matrices in PRIMER 7. To evaluate how latitude affects climate, soil properties, and functional and taxonomic compositions, principal coordinate (PCoA) 1 scores of Euclidean distance of climate and soil data, and Bray-Curtis similarity of function and taxonomy were used to construct Pearson’s correlations with the absolute latitude of soil metagenome locations grouped by 17 climate zones. To assess the associations of functional and taxonomic diversities with climate and soil properties, DistLM (distance-based linear model) (Anderson, 2004) was conducted in PRIMER 7 using forward procedure and adjusted *R*^2^. Pearson’s correlations were further constructed to assess the relationships of specific functional and taxonomic compositions with climate and soil properties. One-way analysis of variance (ANOVA) with *P*-values adjusted by Bonferroni-correction for multiple comparisons was conducted using SPSS 22.0 software (Chicago, IL, United States) to evaluate the statistical differences in the relative abundance of dominant functional and taxonomic compositions (mean > 1%) among climate zones after the normality of residues and homogeneity of variance were checked using Shapiro-Wilk and Levene test, respectively. The significance level was set at α = 0.05 unless otherwise stated. Heat maps were constructed using HeatMapper (Babicki et al., 2016).

To examine the potential interactions of microbial functional and taxonomic compositions across the globe, co-occurrence network analysis was performed using the Molecular Ecological Network Analyses Pipeline^2^ (Zhou et al., 2011; Deng et al., 2012). The data matrix of standardized relative abundance, multiplied by 10^6^ to satisfy the requirements of the pipeline, was uploaded to construct the network with default settings, including (1) only keeping the species present in more than a half of all samples; (2) only filling with 0.01 in blanks with paired valid values; (3) taking logarithm with recommended similarity matrix of Pearson’s correlation coefficient; and (4) calculation ordered to decrease the cutoff from top using regress poisson distribution only. A default cutoff value (similarity threshold, *S*_*t*_) for the similarity matrix was generated to assign a link between the pair of species. Then, the global network properties, the individual nodes’ centrality, and the module separation and modularity were analyzed based on default settings using greedy modularity optimization. Network files were exported and visualized using Cytoscape software (Shannon et al., 2003). The scatter plots of within-module connectivity (Zi) and among-module connectivity (Pi) were constructed to show the network node distribution of module-based topological roles of functional and taxonomic compositions. The threshold values of Zi and Pi for categorizing were 2.5 and 0.62, respectively (Guimerà and Nunes Amaral, 2005; Olesen et al., 2006; Guimerà et al., 2007).

## Results and Discussion

### Weak Association Between Microbial Phylogeny and Functional Potentials

**FIGURE 2:**
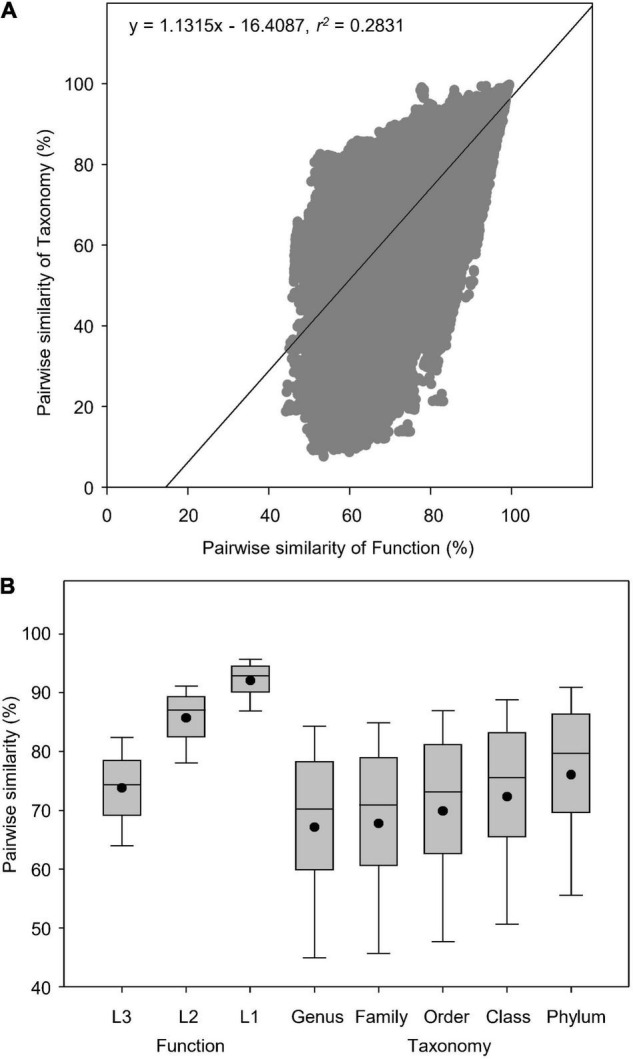
Pairwise similarity of functional and taxonomic diversities. **(A)** Pearson’s correlations between pairwise Bray-curtis similarity of soil metagenomes annotated in Subsystems database at Level 3 (Function) and RefSeq database at genus level (Taxonomy). **(B)** Box plots and mean values of pairwise Bray-curtis similarity of soil metagenomes annotated in the SEED Subsystems database, which are composed of subsystems structured into three levels, i.e., the Level 1 is the most general class; the Level 2 is the more general class; the Level 3 is the most specific class (Function) and RefSeq database at genus, family, order, class, and phylum levels (Taxonomy).

In total, we analyzed 845 soil metagenomes that resulted in 356,090 pairwise comparisons of Bray-curtis similarity in functional (Subsystems Level 3) and taxonomic (RefSeq genus) diversities. Globally, soil taxonomic compositions were more diversely changed than functional potentials (Figures 2A,B). Pearson’s correlation showed that pairwise similarity of function was positively correlated to that of taxonomy as indicated by the linear regression (*r*^2^ = 0.2831) (Figure 2A), which had higher coefficient than the logarithmic regression (*r*^2^ = 0.2752). Paired-samples *t*-test showed that functional potentials had higher similarity (44–99%) than taxonomy (9–100%) (*t* = 67.5, *P* < 0.0001), regardless of the association between functional and taxonomic diversities revealed by Pearson’s correlations (Figure 2A). Functional diversity was more conserved at both classification Level 1 and 2, with higher similarity than taxonomy at all five phylogenetic levels examined (Figure 2B).

Several environmental metagenomic studies have reported linear relationships between functional and taxonomic diversities (Fierer et al., 2012, 2013; Leff et al., 2015), indicating a certain degree of dependency of microbial functional potentials on taxonomic compositions. This significant association, however, does not necessarily imply a lower level of microbial functional redundancy, especially in the terrestrial ecosystems. On the contrary, lower levels of diversity in microbial functional potentials, indicated by the lower dissimilarity in metagenomic composition of functional genes than in taxonomic composition, still supports that the relative abundance of microbial functions based on metagenomic genes are more stable than taxonomy in response to ecological and environmental perturbations. It should be noted that the taxonomic database of RefSeq in our analysis was more diverse than traditional ribosomal RNA databases used in the above mentioned studies. In agreement with our results, some prior studies (Fierer et al., 2012, 2013; Leff et al., 2015) also showed a lower extent of metagenomic diversity in functional potentials compared to taxonomic compositions despite their linear correlations. Our previous study has found a significantly positive correlation between pairwise similarity of function and taxonomy in water metagenomes (*r*^2^ = 0.3344, *P* < 0.0001), though taxonomy had higher global dissimilarity than function (Chen et al., 2021a), suggesting different degrees of microbial function redundancy between soil and water biomes.

### Decoupling Between Microbial and Functional Diversities

**FIGURE 3:**
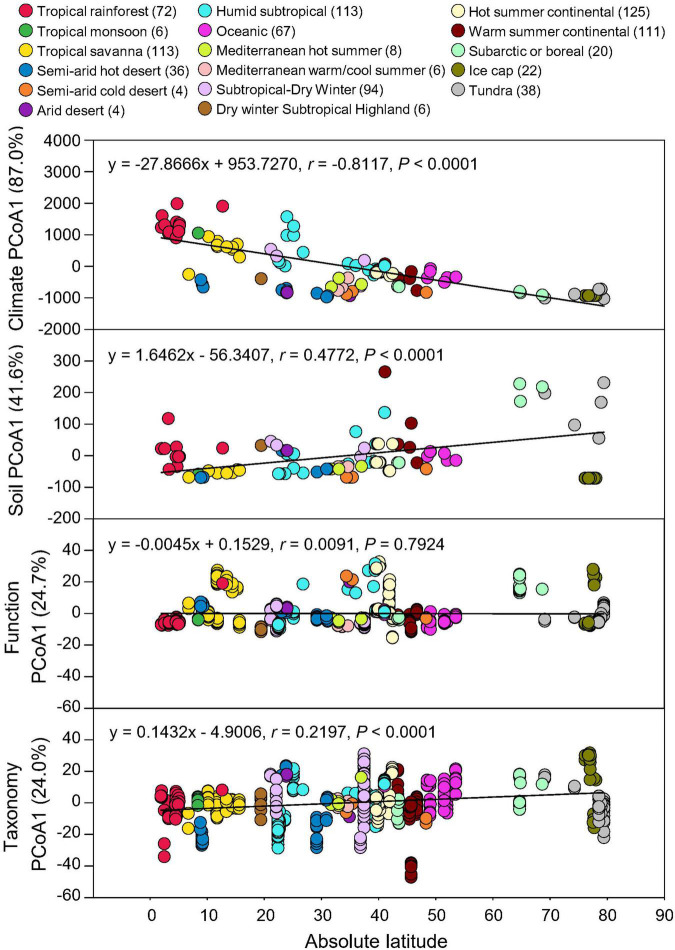
Latitudinal distribution of functional and taxonomic diversities. Pearson’s correlations of the absolute latitude of soil metagenome locations with principal coordinate (PCoA) 1 scores of Euclidean distance of MAT and MAP data (Climate) and soil data of C, pH, sand, silt, and clay (Soil), Bray-curtis similarity of soil metagenomes annotated in the SEED Subsystems database at Level 3 (Function) and RefSeq database at genus level (Taxonomy) grouped by 17 climate zones. Variation explained by principal coordinate dimension is given in parentheses by percentage. Sample sizes of each climate zone classification are given in parentheses. Regression equations, coefficients (*r*), and *P*-values are given.

Absolute latitude of each soil metagenome was used to correlate with climate and soil property data as well as the taxonomic and functional beta-diversities using Pearson’s correlation. Taxonomic compositions showed significant associations with absolute latitudes (*P* < 0.0001), consistent with the latitudinal response of climate (MAT and MAP) and edaphic (SOC, pH, and soil texture) properties, while functional potentials showed no significant latitudinal gradient (*P* = 0.7924) (Figure 3). Thus, geographical/climate gradient could influence soil physicochemical characteristics and microbial taxonomic diversity, but due to functional redundancy, the relative abundances of microbial functional potentials were not significantly affected.

**FIGURE 4:**
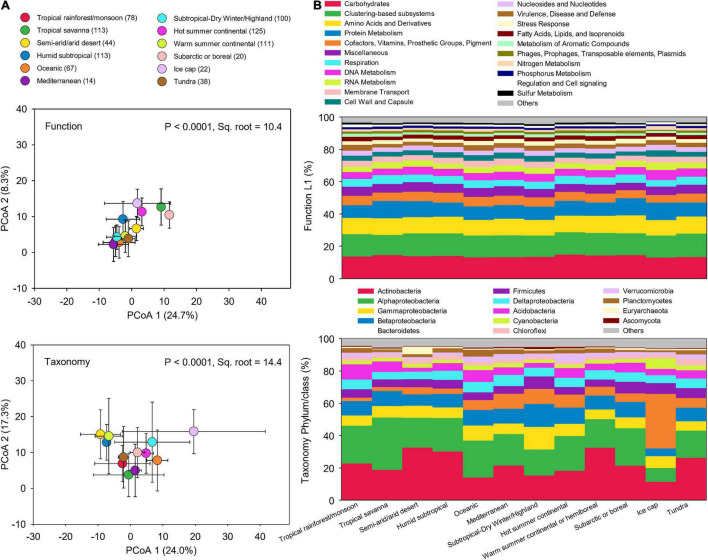
Functional and taxonomic compositions across climate zones. **(A)** Principal coordinates analysis (PCoA) showing beta-diversity of soil metagenomes annotated in the SEED Subsystems database at Level 3 (Function) and RefSeq database at genus level (Taxonomy) grouped by different climate zones. The error bars represent the standard deviation of data ranges. Sample sizes of each climate zone classification are given in parentheses. Variation explained by two principal coordinate dimension is given in parentheses by percentage. *P*-values and Sq. root of PERMANOVA are also given. **(B)** Relative abundance of dominant soil metagenomes (mean > 0.5%) annotated in Subsystems database at level 1 (Function Level 1), and RefSeq database at phylum/class (Taxonomy phylum/class) grouped by different climate zones.

To examine how microbial taxonomic and functional diversities differ globally, soil metagenomic data was were compared among the 17 climate zones. However, some climate zones had very few sites, which may affect the analysis of functional and taxonomic compositions in different climate zones. Thus, we combined the climate zones that have numbers of samples less than 10 based on their upper level classifications, leading to 12 climate zones. Across these climate zones, microbial taxonomic compositions were more dissimilar than functional potentials (Figure 4A). The relative abundance of functional potentials remained stable across climate zones (Figure 4B). In contrast, taxonomic compositions significantly varied across climate zones (Figure 4B). Climate and edaphic properties showed a more significant influence on microbial taxonomic compositions than on functional potentials (Supplementary Table 2). DistLM analysis indicated that the cumulative influences of climate and soil characteristics were also greater on taxonomic compositions (16.3%) than on functional potentials (13.5%) (Supplementary Table 3). The decoupling of soil microbial function and taxonomy has been evaluated in our previous study that soil microbial diversities of taxonomy and function responded distinctly to five levels of simulated reduction of microbial species, leading to stable soil microbial functional structure associated with significant microbial species richness decline (Chen et al., 2021b).

**FIGURE 5:**
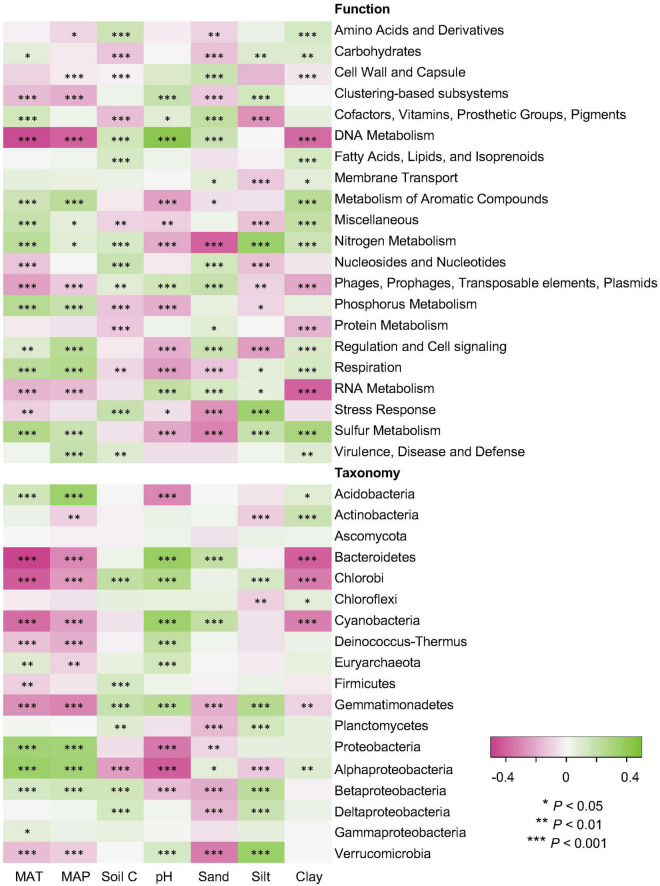
Relations of functional and taxonomic compositions with climate data and soil properties. Heatmaps showing Pearson’s correlations of MAT and MAP data (Climate) and soil properties of C, pH, sand, silt, and clay (Soil) with relative abundance of dominant soil metagenomes (mean > 0.5%) annotated in the SEED Subsystems database at Level 1 (Function) and RefSeq database at phylum/class levels (Taxonomy). The *, ^**^, and ^***^ indicate significant relationship at α = 0.05, 0.01, and 0.001, respectively.

Climate data and soil properties were significantly associated with the relative abundance of dominant functional and taxonomic compositions, as revealed by Pearson’s correlations (Figure 5). For functional potentials, nutrient and energy metabolisms were possitively associated with MAT and MAP, but negatively correlated to soil pH, suggesting an enhanced microbial activity with increasing temperature and precipitation, but limited microbial metabolisms in low pH environments. For taxonomic compositions, soil pH was negatively associated with the abundance/occurrence of Acidobacteria and Proteobacteria, which also indicated opposite trends against the effects of MAT and MAP. It has been found that many Acidobacteria are acidophilic, potentially due to their cell specialization and enzyme stability, and thus more adapted to low pH conditions (Kielak et al., 2016). Proteobacteria, a major phylum of bacteria commonly found in the soil, have a wide variety of metabolism types (Spain et al., 2009), particularly some Alphaproteobacteria that can grow at very low levels of nutrients, perhaps making them dominate in acidic soil environments (Feng et al., 2014). However, it has been shown that Proteobacteria increased in relative abundance with increasing soil pH, especially Alphaproteobacteria that were most abundant in the high pH soils (Rousk et al., 2010). Such contradictory conclusions indicate that additional studies are surely necessary to elucidate the interactions of Proteobacteria and soil pH conditions. In addition, both functional and taxonomic diversities gradually decayed with increasing distance of climate and edaphic properties (Supplementary Figure 1).

However, microbial community structure and functional potentials were solely based on the relative abundance of metagenomic genes, which did not directly involve any microbial functional measurement. In our study, functional potentials relied on the presence of functional genes, but not the interaction of the tested gene pathways with microbial activities and the environment. Thus, it was not concluded that microbial functions were the same across all gradients, but rather many individual metagenomic functional genes, which may or may not be active in a given environment, could be observed in the soil samples across the globe. Latitudinal diversity gradient, a decline of biodiversity with latitude (Hillebrand, 2004), has been found in alpha-diversity of microbes in the soil (Zhou et al., 2016) and marine (Fuhrman et al., 2008; Huerta-Cepas et al., 2015) environments due to temperature and pH gradient. Additionally, we further showed this latitudinal correlation of beta-diversity in microbial taxonomic compositions but not in functional potentials in terrestrial ecosystems.

### Distinct Microbial Co-occurrence Networks Between Function and Taxonomy

**FIGURE 6:**
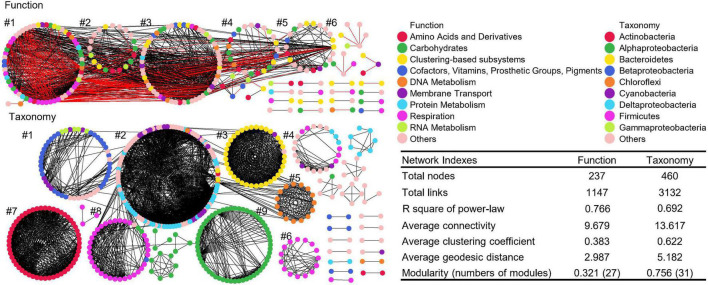
Co-occurrence networks of functional and taxonomic compositions. Network graphs with submodule structures of soil metagenomes annotated in the SEED Subsystems database at Level 3 (Function) and RefSeq database at genus level (Taxonomy). Node color represents classification of Subsystems database at Level 1 or RefSeq database at phylum/class levels. A black edge indicated a positive interaction between two nodes, while a red edge indicated a negative interaction. Summary of key network indexes is given in the table.

The global taxonomic co-occurrence network exhibits higher degrees of modularity than the functional network (Figure 6). Almost all nine major modules of taxonomic network were comprised of genera from the same phylum/class, such as Betaproteobacteria in module #1, Bacteroidetes in module #3, Cyanobacteria in module #5, Gammaproteobacteria in module #6 and 8, Actinobacteria in module #7, and Alphaproteobacteria in module #9. Thus, the interaction of bacteria was conserved in taxonomy, especially at the phylum or class levels. Although the numbers of functional categories (1,036 Level 3) were greater than those of taxonomic groups (885 genus), the functional co-occurrence network had significantly lower degree of modularity, with less total nodes and links, indicating less interactions and connectivity than the taxonomic network, which also potentially infers a certain degree of soil microbial functional redundancy from the perspective of co-occurrence networks. Also, other key network indexes of function and taxonomy, such as the average clustering coefficient, geodesic efficiency, and harmonic geodesic distance were significantly different from those of each other and the corresponding random networks with the same network size and average number of links (Supplementary Table 4).

Despites there are numerous connections among modules of functional networks and within each module, five major modules were identified, and functional nodes could be further classified into multiple categories. Functional network had 805 positive and 342 negative links, suggesting that functional potentials had both facilitative and inhibitive interactions, while taxonomic network only had positive interactions, indicating that the response of taxonomic compositions are all cooperative (Faust and Raes, 2012). Thus, soil microbes from the same groups tended to respond in a similar way, while distinct microorganisms rarely interact with each other, suggesting that the interaction of taxonomic compositions are taxa-specific in niches (Faust and Raes, 2012).

**FIGURE 7:**
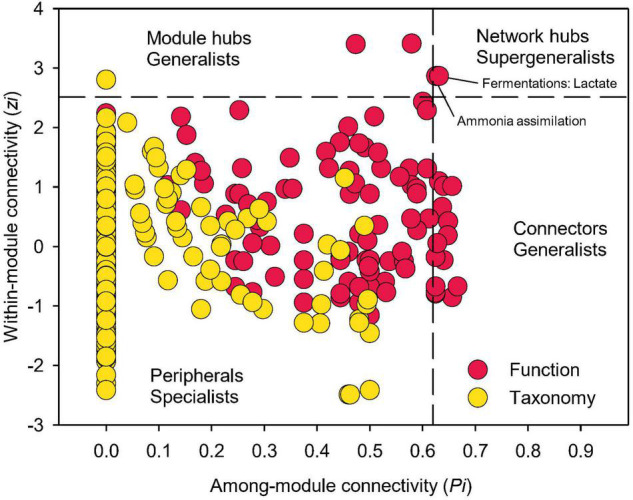
Network information of functional and taxonomic compositions. Node distribution of module-based topological roles determined by the scatter plot of within-module connectivity (*zi*) and among-module connectivity (*Pi*) in soil metagenome networks annotated in the SEED Subsystems database at Level 3 (Function) and RefSeq database at genus level (Taxonomy). The threshold values of Zi and Pi for categorizing were 2.5 and 0.62, respectively.

Examining closely into the co-occurrence network of functional composition, 21 nodes were identified as either module hubs, which were connected to many nodes within their own modules (high *Zi*, low *Pi*), or connectors linking several modules (low *Zi*, high *Pi*), as indicated by the *Zi*-*Pi* plot (Olesen et al., 2007; Deng et al., 2012; Figure 7). Two functional nodes, namely ammonia assimilation and lactate fermentation, were grouped to network hubs as “supergeneralists,” acting as both module hubs and connectors interacting with the rest of the functional network. Significantly less nodes could be identified as module hubs in the co-occurrence network of taxonomic composition, indicating less interactions among different modules. This is expected given that module was comprised of genera that were mainly from the same phylum/class.

Microbial taxonomic composition and diversity plays a critical role in maintaining ecosystem function, and often shows greater sensitivity to environmental disturbance than microbially-mediated functional potentials (Allison and Martiny, 2008). However, taxonomic information alone provides limited utility in predicting biogeochemical transformations or changes of biogeochemical process rates at ecosystem level (Hall et al., 2018). Because of the decoupling of microbial taxonomic composition and functional potentials as we have discussed in the above sections, understanding which members of the microbial community can provide causative linkages would be critical for accurate prediction of microbial metabolic activity and flexibility across space and time. In this study, by analyzing and generalizing microbial taxonomic compositions and functional potentials based on metagenomic analysis of functional genes, we provide a strong evidence for soil microbial functional redundancy across the globe without any direct measurement of microbial functions. The environmental conditions likely determine the microbial taxonomic composition, and microbial phylotypes sharing similar habitat preferences tend to co-occur (Delgado-Baquerizo et al., 2018; Ramírez-Flandes et al., 2019). Though microbial metabolic functions can be strongly coupled to elemental cycles and certain environmental factors, the decoupling of microbial taxonomic compositions and functional potentials is still inevitable when a low-dimensional functional space is projected to a high-dimensional taxonomic space (Louca et al., 2018).

## Concluding Remarks

Investigating functional redundancy with respect to functions associated with elemental cycles provides useful information to guide the development of microbial biogeochemical processes prediction, and further focusing on major pathways of C and N cycles will be a fruitful approach for representing microbes in functional prediction. We emphasize that this analysis is a combination of snapshots of microbial communities compared across space. As environmental conditions (at the same geographic location) vary, the levels of functional redundancy may change depending upon the mechanisms selecting specific functions and the phylogenetic distribution of those functions (Louca et al., 2018). Although there’s a caveat concerning the direct comparison of metagenomic data, the present study demonstrated the use of comparative metagenome and co-occurrence network analysis in generalizing patterns of microbial characteristics regulating biogeochemical cycling of major elements. With the increasing advancement of sequencing techniques and data coverage, future sequencing efforts will likely increase our confidence in comparative metagenomes and provide time-series information to further identify to what extent microbial functional redundancy regulates dynamic ecological fluxes across space and time.

## Data Availability Statement

The original contributions presented in the study are included in the article/Supplementary Material, further inquiries can be directed to the corresponding author/s.

## Author Contributions

HuC conceived the study, performed the data analysis, interpreted the results, and drafted the manuscript. CL, YY, CS, and HaC secured the research funding. KM, CL, YY, and HaC critically assessed and interpreted the findings. All authors discussed results, commented on, edited, revised, and approved the manuscript.

## Conflict of Interest

The authors declare that the research was conducted in the absence of any commercial or financial relationships that could be construed as a potential conflict of interest.

## Publisher’s Note

All claims expressed in this article are solely those of the authors and do not necessarily represent those of their affiliated organizations, or those of the publisher, the editors and the reviewers. Any product that may be evaluated in this article, or claim that may be made by its manufacturer, is not guaranteed or endorsed by the publisher.

## Footnotes

1
https://soilgrids.org/

2
http://ieg4.rccc.ou.edu/MENA/
