# Supplementary material for: Functional Redundancy in Soil Microbial Community Based on Metagenomics Across the Globe
Source: Front Microbiol. 2022 May 2;13:878978. doi: 10.3389/fmicb.2022.878978 (PMC9108720; doi:10.3389/fmicb.2022.878978)

**Supplementary Table 1. Information of MG-RAST soil metagenomes.** Data are extracted from publications used in this study including study ID, MG-RAST ID, sample name, publication, bp, reads, latitude and longitude, climate zone, climate code, mean annual temperature (MAT, °C), mean annual precipitation (MAP, mm yr^-1^), soil C, pH, Sand (%), Silt (%), Clay (%), and hits in Subsystems and RefSeq databases.

Available in a separated Excel spreadsheet file.

**Supplementary Table 2.** **Relations of functional and taxonomic compositions with climate and soil data.** Results of the marginal test performed by DistLM (distance-based linear model) analysis to show the associations of the soil metagenomes annotated in Subsystems at level 3 (Function) and RefSeq at genus level (Taxonomy) with MAT and MAP data (Climate) and soil properties of C, pH, Sand, Silt, and Clay (Soil).

| Variable | SS(trace) | Pseudo-F | P | Prop.^#^ |
| --- | --- | --- | --- | --- |
| Function | |  |  |  |
| MAT | 8557.7 | 23.652 | 0.0001 | 0.0273 |
| MAP | 6807.5 | 18.708 | 0.0001 | 0.0217 |
| C | 5260.0 | 14.382 | 0.0001 | 0.0168 |
| pH | 8251.0 | 22.781 | 0.0001 | 0.0263 |
| Sand | 6102.1 | 16.730 | 0.0001 | 0.0195 |
| Silt | 8535.4 | 23.589 | 0.0001 | 0.0272 |
| Clay | 8745.9 | 24.187 | 0.0001 | 0.0279 |
| Taxonomy | |  |  |  |
| MAT | 29540.0 | 46.492 | 0.0001 | 0.0523 |
| MAP | 24547.0 | 38.278 | 0.0001 | 0.0434 |
| C | 9187.4.0 | 13.931 | 0.0001 | 0.0163 |
| pH | 28666.0 | 45.043 | 0.0001 | 0.0507 |
| Sand | 9473.7 | 14.372 | 0.0001 | 0.0168 |
| Silt | 13314.0 | 20.338 | 0.0001 | 0.0236 |
| Clay | 17644.0 | 27.167 | 0.0001 | 0.0312 |

^#^Prop. denotes the proportion of explained variation.

**Supplementary Table 3. Effects of climate and soil data on functional and taxonomic compositions.** Results of the sequential test performed by DistLM (distance-based linear model) analysis to show how MAT and MAP data (Climate) and soil properties of C, pH, Sand, Silt, and Clay (Soil) together influence the soil metagenomes annotated in Subsystems at level 3 (Function) and RefSeq at genus level (Taxonomy).

| Variable | SS(trace) | Pseudo-F | P | Prop. ^#^ | Cumul. |
| --- | --- | --- | --- | --- | --- |
| Function | |  |  |  |  |
| +MAT | 8745.9 | 24.187 | 0.0001 | 0.0279 | 0.0279 |
| +MAP | 8462.3 | 24.042 | 0.0001 | 0.0270 | 0.0549 |
| +C | 7037.1 | 20.455 | 0.0001 | 0.0224 | 0.0773 |
| +pH | 5758.7 | 17.059 | 0.0001 | 0.0184 | 0.0957 |
| +Sand | 4804.4 | 14.460 | 0.0001 | 0.0153 | 0.1110 |
| +Silt | 4362.3 | 13.322 | 0.0001 | 0.0139 | 0.1249 |
| +Clay | 3068.0 | 9.464 | 0.0001 | 0.0098 | 0.1347 |
| Taxonomy | |  |  |  |  |
| +MAT | 29540.0 | 46.492 | 0.0001 | 0.0523 | 0.0523 |
| +MAP | 13773.0 | 22.222 | 0.0001 | 0.0244 | 0.0766 |
| +C | 12802.0 | 21.150 | 0.0001 | 0.0227 | 0.0993 |
| +pH | 9863.6 | 16.598 | 0.0001 | 0.0175 | 0.1167 |
| +Sand | 8839.6 | 15.125 | 0.0001 | 0.0156 | 0.1324 |
| +Silt | 11357.0 | 19.869 | 0.0001 | 0.0201 | 0.1525 |
| +Clay | 5887.0 | 10.415 | 0.0001 | 0.0104 | 0.1629 |

^#^Prop. denotes the proportion of explained variation; Cumul. means the cumulative proportion of explained variation.

**Supplementary Table 4. Key indexes of functional, taxonomic, and their associated random networks.**

| Network Indexes | Function | Function Random | Taxonomy | Taxonomy Random |
| --- | --- | --- | --- | --- |
| Average clustering coefficient | 0.383 | 0.245 ± 0.011 | 0.622 | 0.064 ± 0.004 |
| Average geodesic distance | 2.987 | 2.739 ± 0.029 | 5.182 | 2.770 ± 0.016 |
| Geodesic efficiency | 0.394 | 0.405 ± 0.003 | 0.283 | 0.396 ± 0.002 |
| Harmonic geodesic distance | 2.54 | 2.469 ± 0.021 | 3.532 | 2.528 ± 0.011 |
| Centralization of degree | 0.253 | 0.253 ± 0.000 | 0.095 | 0.095 ± 0.000 |
| Centralization of betweenness | 0.067 | 0.104 ± 0.012 | 0.13 | 0.030 ± 0.004 |
| Centralization of stress centrality | 0.786 | 0.561 ± 0.056 | 17.422 | 0.244 ± 0.021 |
| Centralization of eigenvector centrality | 0.182 | 0.186 ± 0.006 | 0.193 | 0.122 ± 0.008 |
| Density (D) | 0.041 | 0.041 ± 0.000 | 0.03 | 0.030 ± 0.000 |
| Transitivity (Trans) | 0.489 | 0.302 ± 0.008 | 0.664 | 0.082 ± 0.002 |
| Connectedness (Con) | 0.637 | 0.949 ± 0.026 | 0.453 | 0.982 ± 0.011 |
| Efficiency | 0.941 | 0.961 ± 0.001 | 0.939 | 0.972 ± 0.000 |

**Supplementary figure legends**

**Supplementary Fig. 1. Decay of functional and taxonomic beta-diversities against climate data and soil properties.** Pearson’s correlations of pairwise Bray-curtis similarity of soil metagenomes annotated in the SEED Subsystems database at Level 3 (Function) and RefSeq database at genus level (Taxonomy) with Euclidean distance of MAT and MAP data (Climate) and soil properties of C, pH, sand, silt, and clay (Soil). Correlation *r*-squared is given.

**Supplementary Fig. 1.**


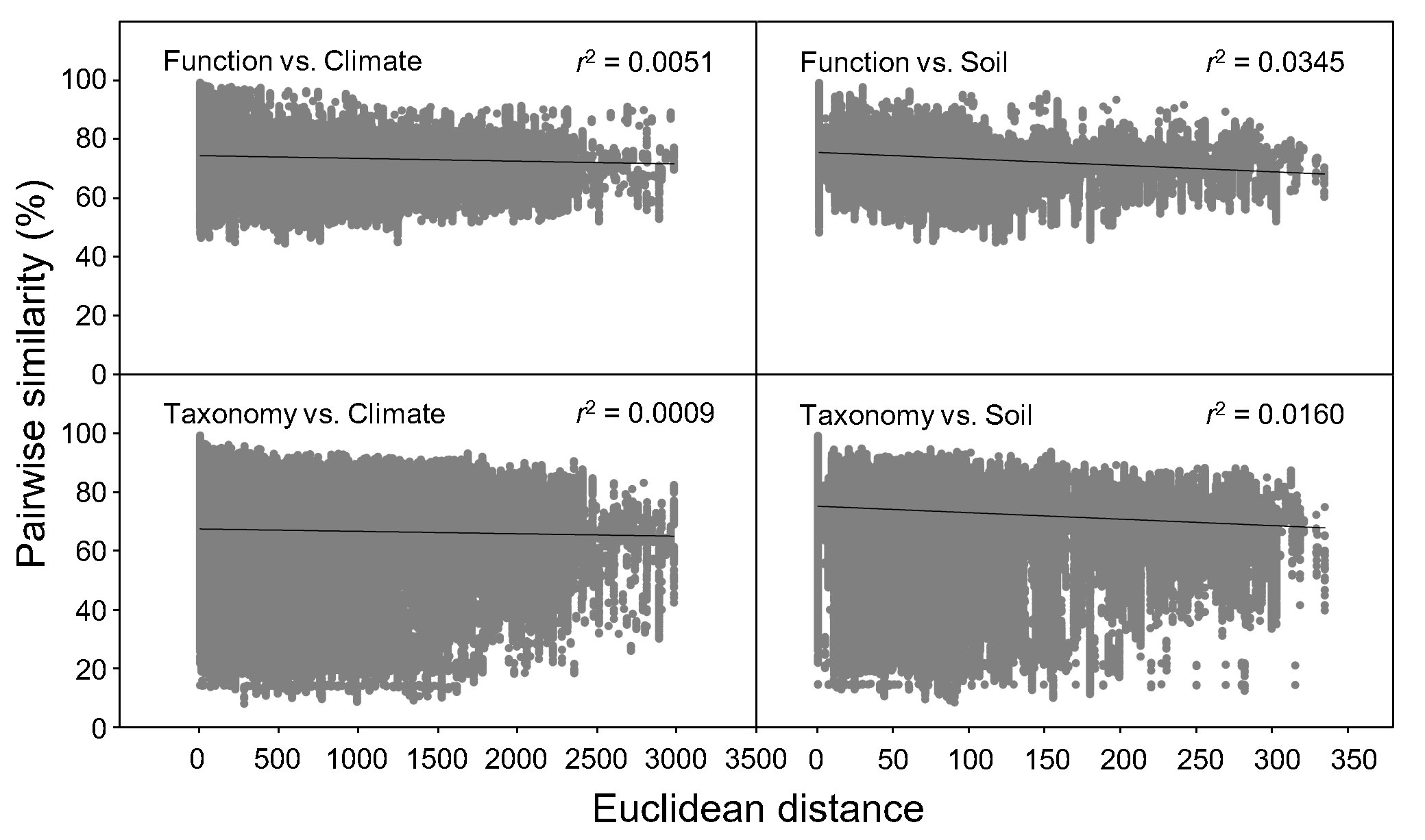

Supplement: Supplementary file 1 [file Data_Sheet_1.docx]
